# Factors influencing the association between depressive symptoms and cardiovascular disease in US population

**DOI:** 10.1038/s41598-024-64274-3

**Published:** 2024-06-13

**Authors:** Keming Ren, Yan Ma, Shuaijie Chen, Peng Wang, Zhezhe Chen, Wuhua Zhang, Yufei Chen, Tianping Zhou, Qianqian Bian, Wenbin Zhang

**Affiliations:** 1 Department of Cardiology, Sir Run Run Shaw Hospital, College of Medicine, Zhejiang University, Hangzhou, 310000 Zhejiang Province China; 2 Key Laboratory of Cardiovascular Intervention and Regenerative Medicine of Zhejiang Province, Hangzhou, 310000 Zhejiang China; 3 Department of Psychiatry, Sir Run Run Shaw Hospital, College of Medicine, Zhejiang University, Hangzhou, 310000 Zhejiang China; 4 Department of Cardiology, The First Affiliated Hospital, Fujian Medical University, Fuzhou, 350000 Fujian China

**Keywords:** Depressive symptoms, Depression, Cardiovascular disease, Cardiovascular risk, NHANES, Depression, Cardiovascular diseases

## Abstract

Cardiovascular disease (CVD) and depression are common diseases that lead to adverse health outcomes. Depressive Symptoms may be a risk factor for CVD. But few studies focused on the impact of socioeconomic factors, common medical history and dietary intake about this association. This study analyzed National Health and Nutrition Examination Survey (NHANES) 2007–2016. Complex sampling-weighted logistic regression models were used to compare the odds ratios (ORs) of CVD in participants with different depressive symptoms. 11,516 NHANES participants aged ≥ 40 years were included in the final analysis, of whom 1842 had CVD. Compared with participants with no/minimal depression, participants with mild, moderate, and moderately severe/severe depression had OR values of 1.25 (95%  CI 1.01–1.54), 1.98 (95% CI 1.32–2.96), and 2.41 (95% CI 1.63–3.57). The association of depressive symptoms with CVD follow a dose-dependent pattern. The interactions of depressive symptoms with gender (Interaction* P* = 0.009), diabetes (Interaction* P* = 0.010), household income level (Interaction* P* = 0.002), dietary cholesterol intake (Interaction* P* = 0.017) on CVD were observed. More severe depressive symptoms are associated with increased risk of CVD in US population. The association may be more pronounced in the female population, population with diabetes, low family income level, or high dietary cholesterol intake.

## Introduction

Cardiovascular disease (CVD) is a group of heart and vascular diseases, mainly represented by ischemic heart disease and stroke, that are the leading causes of death and disability globally. The Global Burden of Disease Study shows that the incidence of CVD is on the rise worldwide, with total cases reaching 523 million and deaths from CVD reaching 18.6 million in 2019^1^. Therefore, it is crucial to recognize and manage CVD risk factors. In addition to traditional risk factors, the impact of psychological factors on CVD has been paid increasingly more attention.

Depression is a prevalent chronic medical condition that affects thought, mood, and physical health, is one of the main causes of disability worldwide^2^. The Global Burden of Disease Study showed depression affected approximately 280 million people and accounted for more than 47 million disability-adjusted life-years in 2019^3^. Multiple studies have reported associations between depression and a variety of CVDs, especially coronary heart disease and stroke^4–11^. There are many common pathogenic mechanisms in depression and CVD, including hypothalamic–pituitary–adrenal axis, autonomic dysfunction, genetic factors, etc^12–17^. Some potential factors may influence the association between depression and CVD through these co-pathogenic mechanisms. However, previous studies have not paid sufficient attention to these potential factors. the association of depression and CVD may be different by these neglected factors, such as common medical history, socioeconomic factors, dietary intake, and lifestyle. It is promising to identify the potential factors influencing the association between depression and CVD for the prevention and treatment of depression and CVD.

The Patient Health Questionnaire depression module (PHQ-9) is a 9-item self-administered instrument used for detecting depression and assessing severity of depression^18,19^. For more than two decades, numerous studies have determined the reliability of PHQ-9 for screening for depression^18–22^. And its simplicity makes PHQ-9 a useful clinical and research tool widely used in primary health care worldwide^21^. This study aimed to investigate the association between depressive symptoms (assessed by PHQ-9) and CVD, paying attention to the impact of common medical history, socioeconomic factors, dietary intake and lifestyle on this association.

## Methods

### Study participants

NHANES is a national survey of the health and nutrition status of the US population. The US National Center for Health Statistics (NCHS) is in charge of generating critical health statistics, and accomplishes this by utilizing a stratified, multi-stage probability sampling design that makes it possible for participants to be an accurate representation of the US civilian deinstitutionalized population^23^. The NCHS Research Ethics Review Board approved the NHANES protocol^24^. Written informed consent was obtained from each participant when NHANES was conducted.

**Figure 1:**
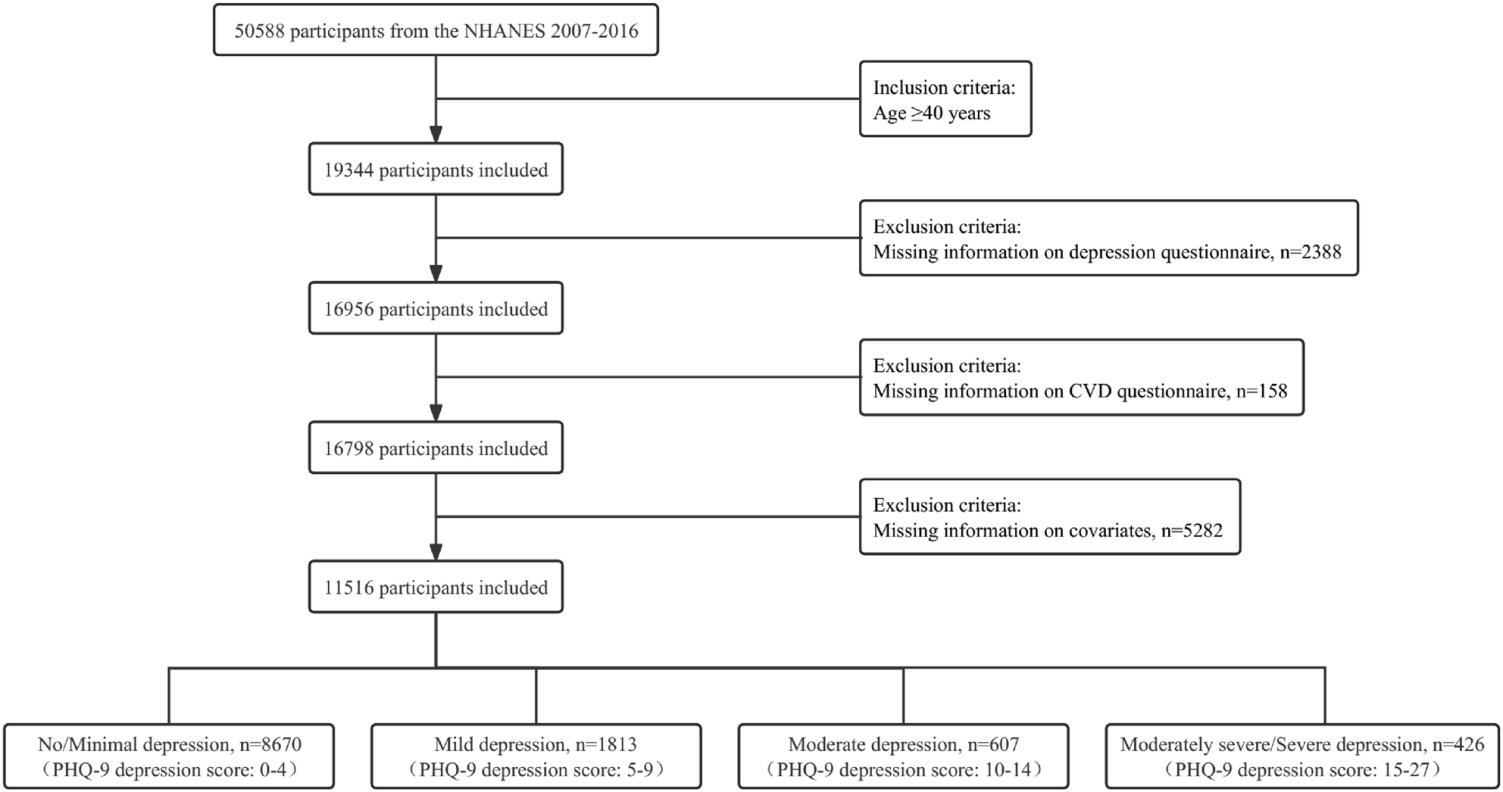
Flowchart of participants selection.

This study obtained data on participants between 2007 and 2016 from the NHANES database. We focused on participants aged 40 years or older at baseline (*n* = 19,344). Since there were few cases with CVD under the age of 40, the 40-year-old cutoff was used in this study. Participants with depression screener and CVD questionnaire data were retained (*n* = 16,798). Then, participants with incomplete covariate information were excluded (*n* = 5282). Finally, A total of 11,516 participants were included in the analysis (Fig. 1).

### Assessment of outcome

CVD was determined using a standardized medical condition questionnaire filled out during the individual interview. The detailed questionnaire and interview procedures are available on the NHANES website^25^. Participants were considered CVD patients if they answered “yes” to any of the following structured questions: Has a doctor or other health professional ever told you that you had congestive heart failure, coronary heart disease, angina (also called angina pectoris), heart attack (also called myocardial infarction), or stroke?

### Assessment of depressive symptoms

Depressive Symptoms was assessed using the Patient Health Questionnaire-9 (PHQ-9). This is a self-reported assessment based on the nine signs and symptoms for depression on Diagnostic and Statistical Manual of Mental Disorders fourth edition. Detailed questionnaires are available from the NHANES. When responding to the nine symptom questions, participants answered according to one of the following categories: “not at all”, “several days”, “more than half the days”, and “nearly every day”. Depression severity can be defined by several cut points from the total score that ranges from 0 to 27: No/Minimal (0–4), mild (5–9), moderate (10–14), moderately severe (15–19) and severe (20–27). Due to the small number of participants with moderately severe or severe depression, they were combined into one group in this study.

### Covariates

Information on age, sex (male, female), race/ethnicity (Hispanic and Mexican American, , non-Hispanic white, non-Hispanic black, or other race), educational level (less than high school, high school graduate/ equivalent, or more than high school), marital status (married/living with partner, widowed/separated/divorced, or never married), family income-poverty ratio (< 1.30, 1.30–3.49, ≥ 3.50), smoking status (current, former, or never), alcohol consumption (< 12 or ≥ 12 alcoholic drinks/year), and disease histories (including trouble sleeping, hypertension, diabetes, dyslipidemia, and cancer) was collected through questionnaires during the survey interviews. Based on the dietary information collected from the 24-h recall interviews, total daily dietary protein, sugar, fiber, cholesterol, caffeine intakes were obtained, and the Healthy Eating Index (HEI) 2010 was used to represent the overall quality of the diet (with scores ranging from 0 to 100, where higher scores represent better dietary quality)^26^. Data of body mass index (BMI), blood pressure, glycohemoglobin, total cholesterol, triglycerides, low-density lipoprotein, high-density lipoprotein, and serum creatinine were measured according to standard protocols. Based on serum creatinine, the estimated glomerular filtration rate (eGFR) was determined using the Chronic Kidney Disease Epidemiology Collaboration algorithm^27^. Hypertension was defined as measured systolic blood pressure (SBP) ≥ 140 mmHg, or/and diastolic blood pressure (DBP) ≥ 90 mmHg, or/and previous diagnosis of hypertension, or/and taking antihypertensive medicine. Diabetes was defined as measured glycohemoglobin level ≥ 6.5%, or/and previous diagnosis of diabetes, or/and taking anti-diabetes medicine. Dyslipidemia was defined as total cholesterol ≥ 200 mg/dL, triglycerides ≥ 150 mg/dL, low-density lipoprotein ≥ 130 mg/dL or high-density lipoprotein ≤ 40 mg/dL in males and ≤ 50 mg/dL in females. Obesity was defined as BMI ≥ 30 kg/m^2^.

### Statistical analysis

Complex survey designs needed be considered because the NHANES samples were not straightforward random samples. Following the recommendations of the US Centers for Disease Control and Prevention^28^, we utilized appropriate weights for each analysis based on the selected variables. Continuous variables are expressed as mean ± standard error (SE), and comparisons between groups were made using Student's *t*-test. Categorical variables are expressed as percentages, and comparisons between groups were made using the chi-squared test. Complex sampling-weighted univariate logistic regression analysis was used to screen for covariables included in subsequent analyses. Complex sampling-weighted multivariate logistic regression models were established for the odds ratios (ORs) and 95% confidence intervals (95% CIs) between depressive symptoms and CVD. Variance inflation factor is used to evaluate the multicollinearity in multivariate logistic regression models. Likelihood ratio tests were used to assess the statistical efficacy of the model. Model 1 was adjusted for age, sex, race/ethnicity, education level, marital status, PIR. Model 2 was further adjusted for smoking status, alcohol consumption, BMI and HEI. Model 3 was further adjusted for disease histories (trouble sleeping, hypertension, diabetes, dyslipidemia, and cancer), blood pressure, glycohemoglobin, low-density lipoprotein, and eGFR. We developed regression models using not only depression severity, but also PHQ-9 score as a continuous variable. Thereafter, subgroup analyses stratified by sex, age, BMI, race/ethnicity, educational level, marital status, smoking status, alcohol consumption, trouble sleeping, hypertension, diabetes, dyslipidemia, cancer, eGFR, PIR, HEI, serum cotinine and dietary protein, sugar, fiber, cholesterol, caffeine intakes were conducted based on model 3. Meanwhile, interactions between PHQ-9 score and the above stratification variables were assessed. Furthermore, restricted cubic spline regressions were used to explore the dose–response association of PHQ-9 score and CVD in subgroups with significant interactions. All statistical analyses were performed using R version 4.1.2 (R Project for Statistical Computing), and *P* < 0.05 was regarded as statistically significant for all tests.

### Ethics approval and consent to participate

The NCHS Research Ethics Review Board approved the NHANES protocol (https://www.cdc.gov/nchs/nhanes/irba98.htm). The NHANES has obtained written informed consent from each participant.

## Results

### Baseline characteristics

**Figure 2:**
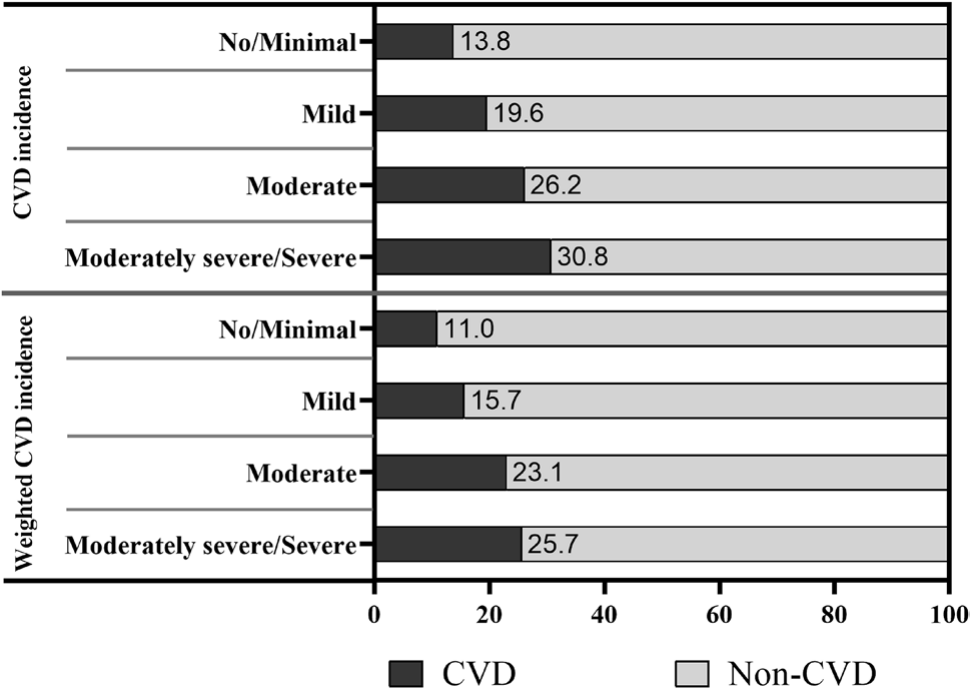
Incidence of CVD in different depressive states. CVD: cardiovascular disease.

In this study, 11516 NHANES participants aged 40 years or older were included in the final analysis, of whom 1842 had CVD. Figure 2 shows the incidence of CVD in different depressive states (including the unweighted and weighted data). Baseline characteristics of patients grouped according to depressive states were listed in Table 1. Participants with more severe depressive symptoms were more likely to be younger, female, non-Hispanic black, Hispanic, non-married, and current smokers, had lower education levels, lower family income level, lower high-density lipoprotein, lower HEI, lower dietary protein, fiber, cholesterol intakes and higher BMI, higher glycohemoglobin, higher triglyceride, higher dietary caffeine intake. In addition, participants with more severe depressive symptoms had higher prevalence of CVD (heart failure, coronary heart disease, stroke, angina, myocardial infarction), trouble sleeping, hypertension, and diabetes.

**Table 1:** Participants baseline demographic and clinical characteristics.

Characteristic	Total	Depressive status				*P* value
		No/Minimal (*n* = 8670)	Mild (*n* = 1813)	Moderate (*n* = 607)	Moderately severe/Severe (*n* = 426)	
Cardiovascular disease, %	12.7	11	15.7	23.1	25.7	< 0.001
Heart failure, %	3.5	2.6	6.1	8	7.8	< 0.001
Coronary heart disease, %	5.5	5.1	5.6	10	8.7	0.005
Stroke, %	4	3.2	5.7	6.9	10.1	< 0.001
Angina, %	3.2	2.5	4.5	5.7	9	< 0.001
Myocardial infarction, %	5	4.5	5.5	8.8	9.5	< 0.001
Age, years	57.97 ± 0.20	58.21 ± 0.24	57.69 ± 0.35	56.55 ± 0.58	55.57 ± 0.58	< 0.001
Gender, %						< 0.001
Male	47.2	50	39.7	33.5	35.1	
Female	52.8	50	60.3	66.5	64.9	
Race, %						< 0.001
Hispanic and Mexican American	9.5	9	10.4	13.3	14.7	
Non-Hispanic White	75.5	76.3	75	66.7	70.2	
Non-Hispanic Black	9.1	8.6	10.4	11.4	11.1	
Other Race	5.9	6.1	4.2	8.7	4	
Education level, %						< 0.001
Less than high school	14.1	12.1	18.7	23.7	27.6	
High school graduation/GED	22.6	21.5	25.9	23	31.2	
More than high school	63.3	66.3	55.4	53.3	41.2	
Marital status, %						< 0.001
Married or living with partner	68.9	72.3	61.5	48.4	51.4	
Widowed, separated or divorced	24.4	21.3	31.6	41.6	40.3	
Never married	6.7	6.4	6.9	10	8.3	
Family income-poverty ratio	3.28 ± 0.05	3.49 ± 0.05	2.83 ± 0.07	2.17 ± 0.13	2.02 ± 0.12	< 0.001
Family income level, %						< 0.001
< 1.30	16.5	12.8	23.3	38.6	41	
1.30–3.49	33.8	32	39.8	38.3	42.7	
≥ 3.50	49.7	55.2	36.9	23.1	16.3	
Smoking status, %						< 0.001
Never	52.4	55.4	46	36.9	32.6	
Former	31.3	31.7	32.3	25.1	27.1	
Current	16.3	12.9	21.7	38	40.2	
Alcohol drinks, %						0.829
Less than 12 alcohol drinks/year	23.8	23.7	24.7	22.6	23.1	
At least 12 alcohol drinks/year	76.2	76.3	75.3	77.4	76.9	
Trouble sleeping, %	33.2	25.8	52.1	66	73.2	< 0.001
Body mass index, kg/m2	29.48 ± 0.12	29.03 ± 0.13	30.76 ± 0.30	31.61 ± 0.50	31.29 ± 0.48	< 0.001
Body mass index, %						< 0.001
< 30	60.4	63.3	52.4	45.7	48.8	
≥ 30	39.6	36.7	47.6	54.3	51.2	
Hypertension, %	51.2	49.2	56.3	60	62.2	< 0.001
Systolic blood pressure, mmHg	125.57 ± 0.30	125.69 ± 0.32	125.49 ± 0.79	125.09 ± 1.51	123.74 ± 1.23	0.439
Diastolic blood pressure, mmHg	71.20 ± 0.25	71.25 ± 0.27	71.08 ± 0.48	70.75 ± 0.73	71.19 ± 0.80	0.902
Diabetes, %	16.6	14.8	21.2	25.1	23.7	< 0.001
Glycohemoglobin	5.81 ± 0.02	5.76 ± 0.02	5.94 ± 0.04	5.97 ± 0.07	5.98 ± 0.07	< 0.001
Dyslipidemia, %	83.3	82.6	86.2	84.8	85.7	0.068
Total cholesterol, mg/dL	200.72 ± 0.79	199.78 ± 0.91	204.05 ± 1.71	204.64 ± 3.39	201.91 ± 3.46	0.101
Triglyceride, mg/dL	166.59 ± 1.64	159.86 ± 2.10	189.70 ± 5.82	186.23 ± 9.82	192.02 ± 10.28	< 0.001
Low-density lipoprotein, mg/dL	113.04 ± 0.65	112.86 ± 0.73	113.28 ± 1.64	115.77 ± 3.05	112.22 ± 3.29	0.816
High-density lipoprotein, mg/dL	54.36 ± 0.30	54.95 ± 0.34	52.83 ± 0.64	51.62 ± 0.79	51.29 ± 0.94	< 0.001
Cancer, %	15.6	15.7	15.5	13.9	14.1	0.844
eGFR	84.65 ± 0.33	84.62 ± 0.37	84.67 ± 0.74	85.39 ± 0.99	84.40 ± 1.37	0.882
eGFR, %						0.371
< 60	10.7	10.4	12.2	10	10.7	
≥ 60	89.3	89.6	87.8	90	89.3	
HEI score	55.88 ± 0.31	56.83 ± 0.32	54.09 ± 0.48	50.53 ± 0.92	49.17 ± 0.98	< 0.001

eGFR, estimated glomerular filtration rate; HEI, Healthy Eating Index.

Continuous variables are expressed as means and standard error and categorical variables as percentages. Means and percentages are weighted.

### Association of depressive symptoms with CVD

Table 2 presented survey-weighted multivariate logistic regression results. Univariate logistic regression analysis was used to screen for covariables included in multivariate logistic regression (Supplementary Table 1). And variance inflation factor is used to evaluate the multicollinearity in the regression model, and variance inflation factor is all less than 5, indicating that the collinearity between variables is reasonable (Supplementary Table 2). Likelihood ratio tests indicated that model 3 had the best statistical performance (Supplementary Table 3). Multivariate adjusted model 3 showed that depressive Symptoms was positively associated with CVD in participants (Supplementary Table 4). Compared with participants with no/minimal depression, participants with mild, moderate, and moderately severe/severe depression had OR values of 1.25 (95% CI, 1.01–1.54), 1.98 (95% CI, 1.32–2.96), and 2.41 (95% CI, 1.63–3.57), respectively. Moreover, the association of depressive symptoms with CVD may be in a dose-dependent manner. When the PHQ-9 depression score was used as a continuous variable, the OR value for CVD was 1.06 (95% CI, 1.04–1.08). In addition, the associations of depressive symptoms with heart failure, coronary heart disease, stroke, angina, and myocardial infarction was similarly significant when assessed separately (Table 2).

**Table 2:** Association between depressive symptoms and cardiovascular disease.

Outcomes	Depressive Status				PHQ-9 as a continuous variable
	No/Minimal	Mild	Moderate	Moderately severe/Severe	
Cardiovascular disease					
Unadjusted OR	1 [Ref]	1.51(1.27–1.80)	2.43(1.80–3.27)	2.80(2.08–3.76)	1.07(1.06–1.09)
*P* value		< 0.001	< 0.001	< 0.001	< 0.001
Model 1 OR	1 [Ref]	1.59(1.32–1.92)	2.81(2.04–3.88)	3.61(2.58–5.06)	1.09(1.07–1.11)
*P* value		< 0.001	< 0.001	< 0.001	< 0.001
Model 2 OR	1 [Ref]	1.45(1.18–1.77)	2.36(1.69–3.30)	3.03(2.14–4.27)	1.08(1.06–1.09)
*P* value		< 0.001	< 0.001	< 0.001	< 0.001
Model 3 OR	1 [Ref]	1.25(1.01–1.54)	1.98(1.32–2.96)	2.41(1.63–3.57)	1.06(1.04–1.08)
*P* value		0.04	0.001	< 0.001	< 0.001
Heart failure					
Unadjusted OR	1 [Ref]	2.45(1.75–3.43)	3.32(2.04–5.39)	3.19(1.88–5.41)	1.09(1.06–1.11)
*P* value		< 0.001	< 0.001	< 0.001	< 0.001
Model 1 OR	1 [Ref]	2.36(1.59–3.49)	3.13(1.91–5.13)	3.31(1.83–5.98)	1.09(1.06–1.11)
*P* value		< 0.001	< 0.001	< 0.001	< 0.001
Model 2 OR	1 [Ref]	2.15(1.44–3.23)	2.68(1.62–4.42)	2.81(1.56–5.08)	1.08(1.05–1.10)
*P* value		< 0.001	< 0.001	< 0.001	< 0.001
Model 3 OR	1 [Ref]	1.78(1.14–2.78)	2.05(1.28–3.29)	1.95(1.11–4.84)	1.05(1.02–1.08)
*P* value		0.012	0.003	0.077	0.001
Coronary heart disease					
Unadjusted OR	1 [Ref]	1.10(0.81–1.51)	2.07(1.24–3.47)	1.77(1.14–2.77)	1.05(1.03–1.06)
*P* value		0.533	0.006	0.012	< 0.001
Model 1 OR	1 [Ref]	1.25(0.90–1.74)	2.86(1.65–4.97)	2.72(1.71–4.33)	1.08(1.06–1.10)
*P* value		0.172	< 0.001	< 0.001	< 0.001
Model 2 OR	1 [Ref]	1.13(0.82–1.58)	2.45(1.35–4.41)	2.28(1.45–3.58)	1.06(1.04–1.09)
*P* value		0.449	0.004	< 0.001	< 0.001
Model 3 OR	1 [Ref]	0.94(0.68–1.29)	1.98(0.95–4.14)	1.81(1.04–3.16)	1.05(1.02–1.08)
*P* value		0.696	0.069	0.037	0.001
Stroke					
Unadjusted OR	1 [Ref]	1.83(1.40–2.39)	2.25(1.39–3.64)	3.39(2.10–5.46)	1.08(1.05–1.11)
*P* value		< 0.001	0.001	< 0.001	< 0.001
Model 1 OR	1 [Ref]	1.67(1.28–2.18)	2.00(1.17–3.40)	3.32(2.02–5.47)	1.08(1.05–1.11)
*P* value		< 0.001	0.012	< 0.001	< 0.001
Model 2 OR	1 [Ref]	1.57(1.20–2.05)	1.75(1.01–3.04)	2.85(1.66–4.88)	1.07(1.04–1.10)
*P* value		0.001	0.045	< 0.001	< 0.001
Model 3 OR	1 [Ref]	1.36(1.05–1.75)	1.46(0.89–2.37)	2.22(1.30–3.79)	1.05(1.02–1.09)
*P* value		0.019	0.13	0.004	0.001
Angina					
Unadjusted OR	1 [Ref]	1.80(1.20–2.72)	2.31(1.41–3.80)	3.82(2.28–6.39)	1.08(1.06–1.11)
*P* value		0.006	0.001	< 0.001	< 0.001
Model 1 OR	1 [Ref]	1.75(1.16–2.66)	2.17(1.30–3.62)	3.80(2.25–6.40)	1.08(1.06–1.11)
*P* value		0.009	0.004	< 0.001	< 0.001
Model 2 OR	1 [Ref]	1.60(1.04–2.45)	1.83(1.07–3.13)	3.21(1.86–5.53)	1.07(1.04–1.10)
*P* value		0.033	0.028	< 0.001	< 0.001
Model 3 OR	1 [Ref]	1.31(0.84–2.04)	1.29(0.72–2.30)	2.24(1.29–3.87)	1.05(1.02–1.08)
*P* value		0.234	0.384	0.005	0.002
Myocardial infarction					
Unadjusted OR	1 [Ref]	1.23(0.93–1.62)	2.04(1.36–3.05)	2.22(1.47–3.37)	1.05(1.03–1.08)
*P* value		0.143	< 0.001	< 0.001	< 0.001
Model 1 OR	1 [Ref]	1.28(0.94–1.75)	2.23(1.53–3.23)	2.69(1.53–3.23)	1.07(1.04–1.09)
*P* value		0.112	< 0.001	< 0.001	< 0.001
Model 2 OR	1 [Ref]	1.16(0.83–1.60)	1.84(1.27–2.66)	2.20(1.33–3.63)	1.05(1.03–1.08)
*P* value		0.377	0.002	0.003	< 0.001
Model 3 OR	1 [Ref]	0.98(0.71–1.37)	1.46(0.93–2.30)	1.70(1.06–2.74)	1.04(1.01–1.06)
*P* value		0.922	0.096	0.029	0.005

OR, odds ratio; Ref, reference; PIR, family income-poverty ratio; BMI, body mass index; eGFR, estimated glomerular filtration rate; HEI, healthy eating index.

Model 1: adjustments for age, sex, race/ethnicity, education level, marital status, PIR.

Model 2: adjustments for model 1 plus smoking status, alcohol consumption, BMI and HEI.

Model 3: adjustments for model 2 plus disease histories (trouble sleeping, hypertension, diabetes, dyslipidemia, and cancer), blood pressure, glycohemoglobin, low-density lipoprotein, and eGFR.

### Subgroup analysis

Subgroup analyses were conducted to investigate the association of depressive symptoms with CVD in different populations. Table 3 shows that the association between depressive symptoms and CVD remains significant in most subgroups. Interestingly, we observed interactions of depressive symptoms with multiple stratification factors (gender, diabetes, Family income-poverty ratio) on CVD. Compared with participants without depression/mild depression, participants with moderate severe/severe depression had an OR value of 2.98 (95% CI, 1.81–4.91) in the female population, whereas the OR value was 1.32 (95% CI, 0.74–2.37) in the male population. Compared with participants without depression/mild depression, participants with moderate severe/severe depression had an OR value of 4.41 (95% CI, 2.19–8.88) in the population with diabetes, whereas the OR value was 1.87 (95% CI, 1.11–3.17) in the population without diabetes. Compared with participants without depression/mild depression, participants with moderate severe/severe depression had an OR value of 2.66 (95% CI, 1.75–4.03) in the population with low family income level, whereas the OR value was 0.93 (95% CI, 0.20–4.39) in the population with high family income level. To further assess the impact of various important nutrients on the association between depression symptoms and CVD, subgroup analyses were performed with dietary protein, sugar, fiber, cholesterol, and caffeine intakes as stratification factors (Supplementary Table 5). Compared with participants without depression/mild depression, participants with moderate severe/severe depression had an OR value of 5.21 (95% CI, 3.06–8.85) in the population with high dietary cholesterol intake, whereas the OR value was 1.36 (95% CI, 0.82–2.26) in the population with low dietary cholesterol intake.

**Table 3:** Association between depressive symptoms and cardiovascular disease based on model 3 stratified by sex, age, BMI, race/ethnicity, educational level, marital status, smoking status, alcohol consumption, trouble sleeping, hypertension, diabetes, dyslipidemia, cancer, eGFR, PIR, HEI, and serum cotinine.

Outcomes	Total	Cases	Depressive status				PHQ-9 as a continuous variable	*P* value for interaction
			No/Minimal	Mild	Moderate	Moderately severe/Severe		
Sex								
Male	5543	1073	1 [Ref]	1.09(0.73–1.64)	1.55(0.85–2.83)	1.32(0.74–2.37)	1.03(1.01–1.06)	0.009
* P* value				0.665	0.153	0.335	0.018	
Female	5973	769	1 [Ref]	1.36(0.96–1.94)	2.24(1.24–4.07)	2.98(1.81–4.91)	1.07(1.05–1.10)	
*P* value				0.083	0.009	< 0.001	< 0.001	
Age								
< 60	5562	402	1 [Ref]	1.19(0.79–1.79)	1.45(0.78–2.71)	2.06(1.18–3.60)	1.05(1.02–1.08)	0.858
*P* value				0.408	0.239	0.012	0.002	
≥ 60	5954	1440	1 [Ref]	1.23(1.00–1.52)	2.58(1.30–5.15)	2.54(1.49–4.32)	1.07(1.04–1.09)	
*P* value				0.048	0.008	< 0.001	< 0.001	
BMI								
< 30	6742	967	1 [Ref]	1.15(0.88–1.51)	2.50(0.99–6.31)	1.63(0.87–3.07)	1.06(1.02–1.09)	0.072
*P* value				0.291	0.053	0.124	< 0.001	
≥ 30	4774	875	1 [Ref]	1.29(0.90–1.83)	1.61(1.01–2.56)	2.87(1.74–4.73)	1.06(1.03–1.08)	
*P* value				0.158	0.046	< 0.001	< 0.001	
White								
Yes	5747	1090	1 [Ref]	1.26(0.97–1.64)	2.43(1.44–4.10)	2.38(1.37–4.13)	1.07(1.04–1.09)	0.600
*P* value				0.082	0.001	0.003	< 0.001	
No	5769	752	1 [Ref]	1.21(0.85–1.71)	1.25(0.78–1.99)	2.21(1.34–3.64)	1.05(1.02–1.07)	
*P* value				0.282	0.353	0.002	< 0.001	
Education								
Less than high school	2626	543	1 [Ref]	1.13(0.78–1.64)	1.76(1.15–2.70)	2.18(1.16–4.12)	1.06(1.03–1.09)	0.313
*P* value				0.520	0.010	0.017	< 0.001	
at least high school	8890	1299	1 [Ref]	1.30(0.98–1.73)	2.12(1.20–3.72)	2.42(1.54–3.79)	1.06(1.04–1.08)	
*P* value				0.069	0.010	< 0.001	< 0.001	
Marital Status (Married)								
Yes	6843	998	1 [Ref]	1.20(0.92–1.56)	1.74(1.01–2.99)	2.43(1.45–4.08)	1.06(1.03–1.08)	0.633
*P* value				0.169	0.047	0.001	< 0.001	
No	4673	844	1 [Ref]	1.30(0.94–1.79)	2.27(1.21–4.27)	2.40(1.22–4.73)	1.06(1.03–1.10)	
*P* value				0.106	0.012	0.012	< 0.001	
Smoking								
Yes	5589	1141	1 [Ref]	1.03(0.80–1.33)	1.49(0.90–2.46)	2.01(1.26–3.22)	1.04(1.02–1.06)	0.155
*P* value				0.825	0.120	0.004	< 0.001	
No	5927	701	1 [Ref]	1.70(1.19–2.41)	3.51(1.48–8.34)	3.86(1.82–8.22)	1.10(1.06–1.15)	
*P* value				0.004	0.005	< 0.001	< 0.001	
Drink								
Yes	8111	1291	1 [Ref]	1.09(0.85–1.41)	1.65(1.05–2.62)	2.40(1.42–4.04)	1.06(1.03–1.08)	0.596
*P* value				0.487	0.032	0.001	< 0.001	
No	3405	551	1 [Ref]	1.63(1.10–2.43)	2.80(0.91–8.58)	2.14(1.03–4.46)	1.06(1.02–1.12)	
*P* value				0.017	0.071	0.042	0.011	
Trouble sleeping								
Yes	3621	781	1 [Ref]	1.13(0.88–1.46)	1.68(1.10–2.57)	2.40(1.32–4.36)	1.05(1.02–1.08)	0.566
*P* value				0.333	0.018	0.005	< 0.001	
No	7895	1061	1 [Ref]	1.35(0.94–1.94)	2.64(1.14–6.12)	2.13(1.04–4.39)	1.07(1.03–1.12)	
*P* value				0.100	0.025	0.040	< 0.001	
Cancer								
Yes	1669	429	1 [Ref]	1.39(0.85–2.30)	1.64(0.81–3.35)	6.01(2.34–15.43)	1.09(1.04–1.14)	0.100
*P* value				0.188	0.167	< 0.001	< 0.001	
No	9847	1413	1 [Ref]	1.20(0.98–1.48)	1.98(1.23–3.21)	2.00(1.33–3.01)	1.05(1.03–1.08)	
*P* value				0.082	0.006	0.001	< 0.001	
Hypertension								
Yes	6636	1485	1 [Ref]	1.39(1.09–1.79)	1.75(1.16–2.64)	2.05(1.26–3.33)	1.06(1.04–1.08)	0.874
*P* value				0.010	0.008	0.005	< 0.001	
No	4880	357	1 [Ref]	0.75(0.43–1.32)	2.55(1.00–6.51)	3.06(1.59–5.89)	1.06(1.02–1.10)	
*P* value				0.310	0.049	0.001	0.004	
Diabetes								
Yes	2586	716	1 [Ref]	1.50(0.98–2.30)	1.93(1.14–3.25)	4.41(2.19–8.88)	1.08(1.05–1.12)	0.010
*P* value				0.062	0.015	< 0.001	< 0.001	
No	8930	1126	1 [Ref]	1.16(0.90–1.49)	1.99(1.10–3.61)	1.87(1.11–3.17)	1.05(1.03–1.07)	
*P* value				0.236	0.023	0.020	< 0.001	
Dyslipidemia								
Yes	9530	1621	1 [Ref]	1.17(0.92–1.50)	1.97(1.22–3.17)	2.34(1.47–3.70)	1.06(1.03–1.08)	0.650
*P* value				0.203	0.006	< 0.001	< 0.001	
No	1986	221	1 [Ref]	1.92(1.22–3.04)	1.64(0.73–3.71)	3.15(1.07–9.27)	1.08(1.03–1.13)	
*P* value				0.006	0.227	0.037	< 0.001	
eGFR								
< 60	1544	582	1 [Ref]	1.80(1.18–2.75)	1.50(0.78–2.91)	1.13(0.50–2.56)	1.05(1.02–1.09)	0.839
*P* value				0.007	0.221	0.758	0.004	
≥ 60	9972	1260	1 [Ref]	1.10(0.83–1.46)	2.01(1.23–3.27)	2.76(1.87–4.08)	1.06(1.04–1.09)	
*P* value				0.503	0.006	< 0.001	< 0.001	
Family income level								
Low and middle income	6696	1307	1 [Ref]	1.43(1.11–1.83)	2.06(1.27–3.34)	2.66(1.75–4.03)	1.07(1.05–1.09)	0.002
*P* value				0.006	0.004	< 0.001	< 0.001	
High income	4820	535	1 [Ref]	0.88(0.59–1.30)	2.12(0.82–5.47)	0.93(0.20–4.39)	1.02(0.98–1.07)	
*P* value				0.502	0.117	0.924	0.233	
HEI score								
< 50	4137	747	1 [Ref]	0.77(0.55–1.07)	2.01(1.08–3.74)	1.59(0.91–2.78)	1.04(1.01–1.08)	0.707
*P* value				0.120	0.029	0.102	0.014	
≥ 50	7379	1095	1 [Ref]	1.66(1.26–2.19)	1.71(1.11–2.64)	3.50(1.90–6.46)	1.08(1.06–1.11)	
*P* value				< 0.001	0.016	< 0.001	< 0.001	

CVD, cardiovascular disease; Q, quartile; Ref, reference; PIR, family income-poverty ratio; BMI, body mass index; eGFR, estimated glomerular filtration rate; HEI, healthy eating index.

Model 3: adjustments for age, sex, race/ethnicity, education level, marital status, PIR, smoking status, alcohol consumption, BMI, HEI, disease histories (trouble sleeping, hypertension, diabetes, dyslipidemia, and cancer), blood pressure, glycohemoglobin, low-density lipoprotein, and eGFR.

*P* value for interaction: interaction of stratified variable and PHQ-9 score on CVD.

The white race was defined as non-Hispanic white. Low family income level is defined as PIR lower than 3.50. High family income level is defined as PIR of at least 3.50.

### Restricted cubic spline analysis

**Figure 3:**
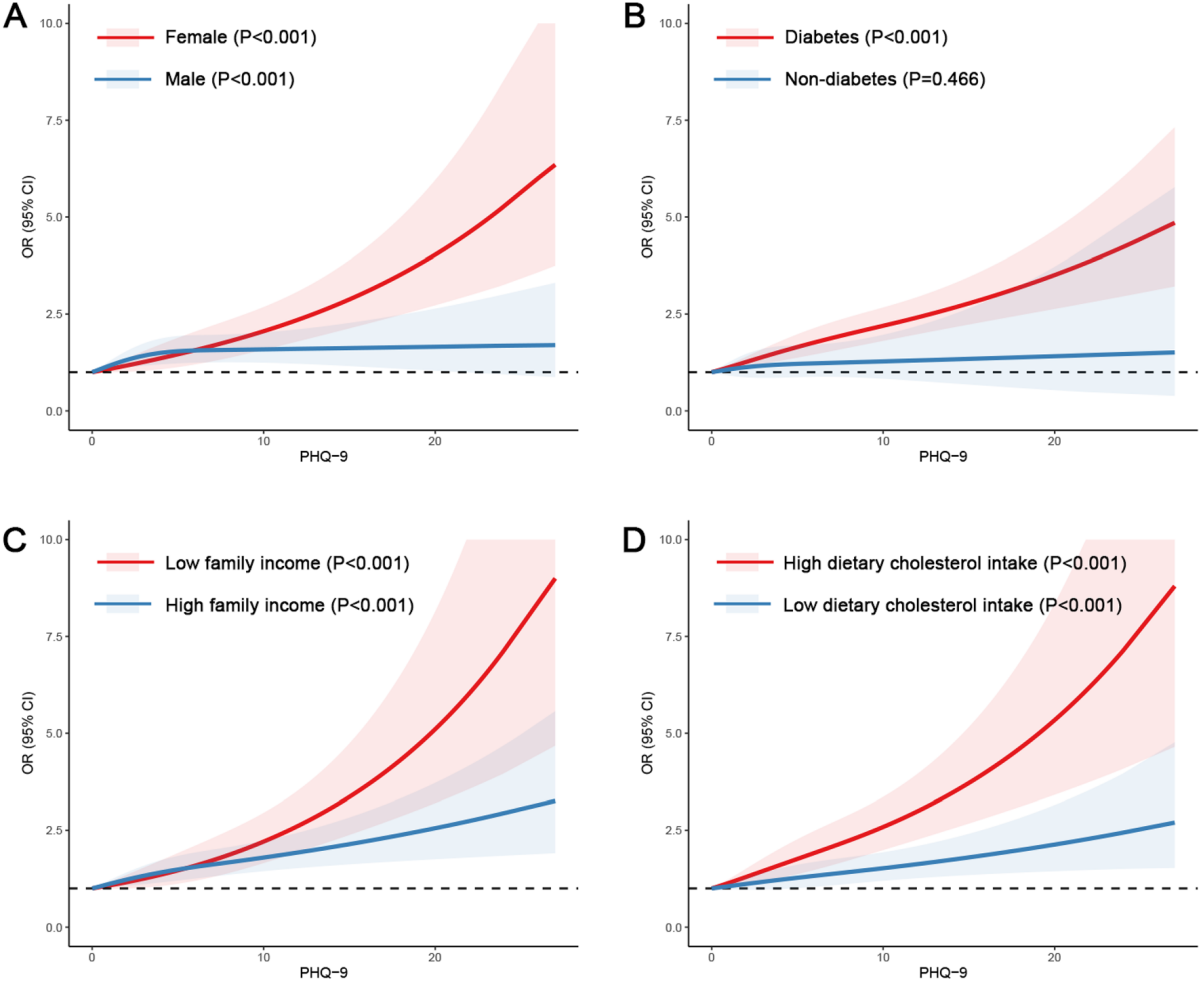
The OR of CVD with PHQ-9 score stratified by gender, diabetes, family income level, and dietary cholesterol intake. OR: Odds ratio; CI: Confidence interval. CVD: cardiovascular disease; PHQ-9: Patient Health Questionnaire-9 (depression score).

The results of restricted cubic spline analysis showed the dose–response association of PHQ-9 score and CVD in subgroups with significant interactions. Figure 3A shows that the dose–response association of PHQ-9 score and CVD were more pronounced in the female population than in the male population. Figure 3B shows that the dose–response association of PHQ-9 score and CVD were more pronounced in the population with diabetes than in the population without diabetes. Figure 3C shows that the dose–response association of PHQ-9 score and CVD were more pronounced in the population with low family income level than in the population with high family income level. Figure 3D shows that the dose–response association of PHQ-9 score and CVD were more pronounced in the population with high dietary cholesterol intake than in the population with low dietary cholesterol intake.

### Sensitivity analysis

Sensitivity analyses were performed to test the robustness of the results, including removing some NHANES data and modifying several covariates. The association between depressive symptoms and CVD was significantly positive in multiple sensitivity analysis results (Supplementary Table 6).

## Discussion

Our study found a significant positive association between depressive symptoms and CVD, even after adjusting for socioeconomic factors, common medical history, and dietary intake. Compared to participants with No/Minimal depressive symptoms, participants with Moderate depressive symptoms had a 98% increased risk of CVD, Participants with Moderately severe/Severe depressive symptoms had a 141% increased risk of CVD. When compared with other risk factors in regression models, the effect of even Moderate depressive symptoms on CVD risk exceeded that of hypertension, diabetes, or dyslipidemia. And there may be a positive dose–response curve between depressive symptoms and CVD. Moreover, the association of depressive symptoms and CVD may be more pronounced in the female population, population with diabetes, population with low family income level, or population with high dietary cholesterol intake.

Some previous studies have investigated the association of depression with CVDs. Multiple meta-analyses have shown a substantial increase (60–80%) in the risk of coronary heart disease associated with depression^5^. In addition, Meijer et al. conducted a meta-analysis of over 25 years of research into the relationship between post-myocardial infarction depression and cardiac prognosis and found that depression was consistently associated with worse prognosis after myocardial infarction^29^. In a pooled analysis of 563 255 participants in 22 cohorts, Harshfield et al. found that baseline depressive symptoms were associated with CVD incidence, including at symptom levels lower than the threshold indicative of a depressive disorder^4^. Similarly, a recent meta-analysis suggests that depression has a significant negative impact on CVD development and CVD outcomes^30^. Several other studies based on the NHANES database have suggested an association between depressive symptoms and cardiovascular mortality^31–33^. However, the current findings suggest that the association between depressive symptoms and CVD risk cannot be explained primarily by established cardiovascular risk factors, including blood pressure, serum lipids, obesity, and diabetes. And few studies have further explored the factors that influence the association between depressive symptoms and CVD. Xu et al. found that Moderate recreational activity modified and mediated the associations between depressive symptoms and CVD based on NHANES 2007–2016^34^. Lu et al. discovered an interaction effect of depressive symptoms and inflammation on the occurrence of CVDs based on NHANES 2007–2016^35^. The factors that influence the association between depression and CVD are not well investigated. Therefore, our study further included some potential influencing factors such as sleep problems, socioeconomic status, multiple dietary intakes, etc., which were not fully considered in previous studies. And our study investigated the influence of these factors on the association between depressive symptoms and CVD. Notably, in addition to gender and diabetes, family income level and dietary cholesterol intake were found to possibly influence the association between depression and CVD. This suggests that the population with low family income level or high dietary cholesterol intake may pay more attention to screening for depression and CVD. This is a promising direction worthy of further research.

For the association between depression and CVD, some possible mechanisms have been proposed. There are data on depression affecting the autonomic nervous system, endothelial function, neurohormonal changes, platelet receptors and function, clotting factors, and pro-inflammatory cytokines, etc^36–39^. The association between depression and CVD may be potentially gender-differentiated in this study, and several previous studies have reported similar results^40–42^. This may be related to sex hormone secretion (e.g., estrogen and progesterone) and related neurotransmitters^43,44^. Current studies have shown that abnormal stress of depression activates the hypothalamic–pituitary–adrenal (HPA) by increasing the concentration of glucocorticoids, which promotes the occurrence and development of cardiovascular diseases^45^. Sex hormones play an important role in the regulation of the HPA axis^46^, which may be the underlying physiological mechanism of the sex difference between depression and cardiovascular diseases. In addition, specific environmental exposures and social processes specifically influence gender differences, involving nutrition in daily life, cultural behaviors, stress responses, and disease prevention^47,48^. Further studies are necessary to explore the underlying specific mechanisms. We also found that diabetes may have affected the association between depression and CVD, which is consistent with previous studies^49^. However, the exact role of diabetes in this process remains unclear. There is evidence that depression and type 2 diabetes share biological origins, particularly overactivation of innate immunity leading to a cytokine-mediated inflammatory response, and potentially through dysregulation of the HPA axis^50^. This may lead to a potential interaction between depression and diabetes on CVD. Our study also found that low family income level and high dietary cholesterol intake may be enhanced the association between depression and CVD. Inoue et al. have suggested that the HPA axis, which is essential for regulating glucocorticoid production by the adrenal glands, may be prone to dysregulation with low socioeconomic status and poor health behaviors^49^. Sullivan et al. proposed that socioeconomic disadvantage can lead to disparities in access, treatment, and care, which may also increase cardiovascular risk through shared stress pathophysiology with depression^51^. Further direct evidence is needed to confirm the effect of socioeconomic status and dietary intake on the association between depression and CVD.

This study focuses on the impact of socioeconomic factors, common medical history, and dietary intake on the association between depressive symptoms and CVD, which provides a reference for the prevention and treatment of depression and CVD in susceptible population. But we also recognize several shortcomings. Depressive symptoms were assessed by a single PHQ-9 depression score and may not fully reflect participants' depressive status. And PhQ-9 does not represent clinical diagnosis. Next, CVD was determined based on the participants' self-reported history of disease, which may have some bias. Finally, this study is cross-sectional. The cross-sectional analysis does not show directionality and we cannot infer if depression leads to CVD or vice versa. Further carefully designed prospective studies are needed to confirm the results of this study.

## Conclusion

More severe depressive symptoms are associated with increased risk of CVD in the US population. The association of depressive symptoms and CVD may be more pronounced in the female population, population with diabetes, population with low family income level, or population with high dietary cholesterol intake.

### Supplementary Information

Supplementary Table 1.

Supplementary Table 2.

Supplementary Table 3.

Supplementary Table 4.

Supplementary Table 5.

Supplementary Table 6.

## Data Availability

The data used in this study are openly available in the NHANES website: NHANES Questionnaires, Datasets, and Related Documentation (https://wwwn.cdc.gov/nchs/nhanes/Default.aspx).
