# Supplementary material for: Factors influencing the association between depressive symptoms and cardiovascular disease in US population
Source: Sci Rep. 2024 Jun 13;14:13622. doi: 10.1038/s41598-024-64274-3 (PMC11176288; doi:10.1038/s41598-024-64274-3)
Supplement: Supplementary file 1 — Supplementary Table 1. [file 41598_2024_64274_MOESM1_ESM.docx]

**Supplementary table 1. Univariate logistic regression analysis for covariables and CVD.**

| **Variables** | **Estimate** | **SE** | **OR (95% CI)** | **P value** |
| --- | --- | --- | --- | --- |
| Age | 0.076 | 0.003 | 1.08(1.07-1.09) | <0.001 |
| Female | -0.421 | 0.078 | 0.66(0.56-0.77) | <0.001 |
| Race |  |  |  |  |
| Non-Hispanic White | 0.334 | 0.099 | 1.40(1.15-1.70) | <0.001 |
| Non-Hispanic Black | 0.450 | 0.105 | 1.57(1.27-1.09) | <0.001 |
| Other Race | 0.265 | 0.191 | 1.30(0.56-0.77) | <0.001 |
| Education level |  |  |  |  |
| High school graduation/GED | -0.330 | 0.104 | 0.72(0.58-0.88) | 0.002 |
| More than high school | -0.778 | 0.096 | 0.46(0.38-0.56) | <0.001 |
| Marital Status |  |  |  |  |
| Widowed, separated or divorced | 0.475 | 0.073 | 1.61(1.39-1.86) | <0.001 |
| Never married | -0.104 | 0.179 | 0.90(0.63-1.29) | 0.562 |
| Family income-poverty ratio | -0.286 | 0.023 | 0.75(0.72-0.79) | <0.001 |
| Smoking status |  |  |  |  |
| Former | 0.624 | 0.092 | 1.87(1.55-2.24) | <0.001 |
| Current | 0.686 | 0.121 | 1.99(1.56-2.53) | <0.001 |
| Alcohol drinks | -0.196 | 0.091 | 0.82(0.68-0.99) | 0.035 |
| Body mass index | 0.032 | 0.005 | 1.03(1.02-1.04) | <0.001 |
| HEI score | -0.008 | 0.002 | 0.99(0.99-0.99) | <0.001 |
| Trouble sleeping | 0.470 | 0.08 | 1.60(1.37-1.88) | <0.001 |
| Hypertension | 1.317 | 0.095 | 3.73(3.09-4.51) | <0.001 |
| Systolic blood pressure | 0.011 | 0.003 | 1.01(1.01-1.02) | <0.001 |
| Diastolic blood pressure | -0.034 | 0.003 | 0.97(0.96-0.97) | <0.001 |
| Diabetes | 1.134 | 0.081 | 3.11(2.65-3.65) | <0.001 |
| Glycohemoglobin | 0.312 | 0.027 | 1.37(1.29-1.44) | <0.001 |
| Dyslipidemia | 0.611 | 0.115 | 1.84(1.47-2.32) | <0.001 |
| Low-density lipoprotein | -0.016 | 0.001 | 0.98(0.98-0.99) | <0.001 |
| Cancer | 0.677 | 0.108 | 1.97(1.59-2.44) | <0.001 |
| eGFR | -0.038 | 0.002 | 0.96(0.96-0.97) | <0.001 |
| CVD, cardiovascular disease; SE, standard error; OR, odds ratio; CI, confidence interval; eGFR, estimated glomerular filtration rate; HEI, healthy eating index. | | | | |
