# Supplementary material for: Factors influencing the association between depressive symptoms and cardiovascular disease in US population
Source: Sci Rep. 2024 Jun 13;14:13622. doi: 10.1038/s41598-024-64274-3 (PMC11176288; doi:10.1038/s41598-024-64274-3)
Supplement: Supplementary file 2 — Supplementary Table 2. [file 41598_2024_64274_MOESM2_ESM.docx]

| **Variables** | **Variance inflation factor** |
| --- | --- |
| PHQ-9 score | 1.916431 |
| Age | 3.242641 |
| Gender | 3.348481 |
| Non-Hispanic White | 3.15941 |
| Non-Hispanic Black | 2.280676 |
| Other race | 1.822274 |
| High school graduation/GED | 2.780376 |
| More than high school | 4.524554 |
| Family income-poverty ratio | 2.846576 |
| Widowed, separated or divorced | 3.1566 |
| Never married | 1.770711 |
| Body mass index | 3.101665 |
| Former smoking | 1.828738 |
| Current smoking | 3.119197 |
| Alcohol drinks | 1.978464 |
| HEI score | 2.107509 |
| Cancer | 2.460483 |
| Hypertension | 1.89081 |
| Systolic blood pressure | 3.897596 |
| Diastolic blood pressure | 2.612263 |
| Diabetes | 3.635452 |
| Glycohemoglobin | 2.766998 |
| eGFR | 2.665915 |
| Dyslipidemia | 1.541932 |
| Low-density lipoprotein | 1.933954 |
| Trouble sleeping | 1.592965 |
| Variance inflation factor is used to evaluate the multicollinearity in the regression model, and variance inflation factor is all less than 5, indicating that the collinearity between independent variables is reasonable | |

**Supplementary table 2. Assessment of multicollinearity for regression model 3.**
