# Supplementary material for: Factors influencing the association between depressive symptoms and cardiovascular disease in US population
Source: Sci Rep. 2024 Jun 13;14:13622. doi: 10.1038/s41598-024-64274-3 (PMC11176288; doi:10.1038/s41598-024-64274-3)
Supplement: Supplementary file 3 — Supplementary Table 3. [file 41598_2024_64274_MOESM3_ESM.docx]

**Supplementary table 3. Evaluation of model efficacy by likelihood ratio test.**

| **Model** | **r^2^** | **AIC** | **logLR** | **P value (LRT)** |
| --- | --- | --- | --- | --- |
| Unadjusted | 0.02 | 8668.92 |  |  |
| Model 1 | 0.21 | 7456.43 | 777.02 (Compare with unadjusted model) | <0.001 |
| Model 2 | 0.24 | 7303.54 | 87.47 (Compare with model 1) | <0.001 |
| Model 3 | 0.30 | 6864.52 | 235.01 (Compare with model 2) | <0.001 |
| AIC, Akaike's An Information Criterion; logLR, Log-Likelihood Ratio; LRT, likelihood ratio tests.  Model 1: adjustments for age, sex, race/ethnicity, education level, marital status, PIR.  Model 2: adjustments for model 1 plus smoking status, alcohol consumption, BMI and HEI.  Model 3: adjustments for model 2 plus disease histories (trouble sleeping, hypertension, diabetes, dyslipidemia, and cancer), blood pressure, glycohemoglobin, low-density lipoprotein, and eGFR. | | | | |
