# Supplementary material for: Factors influencing the association between depressive symptoms and cardiovascular disease in US population
Source: Sci Rep. 2024 Jun 13;14:13622. doi: 10.1038/s41598-024-64274-3 (PMC11176288; doi:10.1038/s41598-024-64274-3)
Supplement: Supplementary file 4 — Supplementary Table 4. [file 41598_2024_64274_MOESM4_ESM.docx]

**Supplementary table 4. Results of complex sampling-weighted multivariate logistic regression model 3.**

|  | Estimate | Standard error | t value | OR | 95% CI | P value |
| --- | --- | --- | --- | --- | --- | --- |
| Mild depressive symptoms | 0.220 | 0.105 | 2.104 | 1.25 | 1.01-1.54 | 0.040 |
| Moderate depressive symptoms | 0.683 | 0.200 | 3.414 | 1.98 | 1.32-2.96 | 0.001 |
| Moderately severe/Severe depressive symptoms | 0.880 | 0.196 | 4.500 | 2.41 | 1.63-3.57 | <0.001 |
| Age | 0.065 | 0.005 | 12.668 | 1.07 | 1.06-1.08 | <0.001 |
| Gender | -0.591 | 0.106 | -5.598 | 0.55 | 0.45-0.68 | <0.001 |
| Non-Hispanic White | 0.234 | 0.119 | 1.967 | 1.26 | 1.00-1.60 | 0.055 |
| Non-Hispanic Black | 0.392 | 0.121 | 3.236 | 1.48 | 1.16-1.89 | 0.002 |
| Other race | 0.393 | 0.215 | 1.823 | 1.48 | 0.96-2.28 | 0.074 |
| High school graduation/GED | -0.059 | 0.115 | -0.514 | 0.94 | 0.75-1.19 | 0.609 |
| More than high school | -0.129 | 0.120 | -1.077 | 0.88 | 0.69-1.12 | 0.286 |
| Family income-poverty ratio | -0.156 | 0.030 | -5.159 | 0.86 | 0.80-0.91 | <0.001 |
| Widowed, separated or divorced | 0.021 | 0.105 | 0.205 | 1.02 | 0.83-1.26 | 0.839 |
| Never married | -0.003 | 0.208 | -0.014 | 1.00 | 0.66-1.51 | 0.989 |
| Body mass index | 0.024 | 0.007 | 3.358 | 1.02 | 1.01-1.04 | 0.001 |
| Former smoking | 0.191 | 0.088 | 2.164 | 1.21 | 1.01-1.45 | 0.035 |
| Current smoking | 0.830 | 0.164 | 5.073 | 2.29 | 1.65-3.19 | <0.001 |
| Alcohol drinks | -0.029 | 0.112 | -0.261 | 0.97 | 0.78-1.22 | 0.795 |
| HEI score | -0.002 | 0.003 | -0.695 | 1.00 | 0.99-1.00 | 0.490 |
| Cancer | 0.059 | 0.133 | 0.445 | 1.06 | 0.81-1.39 | 0.658 |
| Hypertension | 0.624 | 0.110 | 5.670 | 1.87 | 1.50-2.33 | <0.001 |
| Systolic blood pressure | -0.010 | 0.003 | -2.893 | 0.99 | 0.98-1.00 | 0.006 |
| Diastolic blood pressure | -0.004 | 0.004 | -1.028 | 1.00 | 0.99-1.00 | 0.309 |
| Diabetes | 0.265 | 0.125 | 2.121 | 1.30 | 1.01-1.68 | 0.039 |
| Glycohemoglobin | 0.062 | 0.041 | 1.505 | 1.06 | 0.98-1.16 | 0.139 |
| eGFR | -0.014 | 0.002 | -6.002 | 0.99 | 0.98-0.99 | <0.001 |
| Dyslipidemia | 0.608 | 0.133 | 4.563 | 1.84 | 1.41-2.40 | <0.001 |
| Low-density lipoprotein | -0.010 | 0.001 | -7.244 | 0.99 | 0.99-0.99 | <0.001 |
| Trouble sleeping | 0.299 | 0.103 | 2.914 | 1.35 | 1.10-1.66 | 0.005 |
| OR, odds ratio; CI, confidence interval; eGFR, estimated glomerular filtration rate; HEI, healthy eating index. | | | | | | |
