# Supplementary material for: Factors influencing the association between depressive symptoms and cardiovascular disease in US population
Source: Sci Rep. 2024 Jun 13;14:13622. doi: 10.1038/s41598-024-64274-3 (PMC11176288; doi:10.1038/s41598-024-64274-3)
Supplement: Supplementary file 5 — Supplementary Table 5. [file 41598_2024_64274_MOESM5_ESM.docx]

**Supplementary table 5. Association between depressive symptoms and cardiovascular disease based on model 3 stratified by dietary protein, sugar, fiber, cholesterol, caffeine intakes.**

| Outcomes | Total | Cases | Depressive Status | | | | PHQ-9 as a continuous variable | P value for interaction |
| --- | --- | --- | --- | --- | --- | --- | --- | --- |
|  |  |  | No/Minimal | Mild | Moderate | Moderately severe/Severe |  |  |
| Dietary protein intake | |  |  |  |  |  |  |  |
| low | 5758 | 1061 | 1 [Ref] | 1.10(0.87-1.40) | 1.54(0.82-2.88) | 1.95(1.15-3.34) | 1.04(1.01-1.07) | 0.076 |
| P value |  |  |  | 0.422 | 0.172 | 0.015 | 0.003 |  |
| high | 5758 | 781 | 1 [Ref] | 1.43(1.04-1.97) | 2.84(1.72-4.68) | 3.15(1.85-5.36) | 1.09(1.06-1.12) |  |
| P value |  |  |  | 0.028 | <0.001 | <0.001 | <0.001 |  |
| Dietary sugar intake | |  |  |  |  |  |  |  |
| low | 5757 | 994 | 1 [Ref] | 1.58(1.21-2.06) | 1.92(1.27-2.91) | 3.33(1.75-6.35) | 1.07(1.05-1.10) | 0.11 |
| P value |  |  |  | 0.001 | 0.003 | <0.001 | <0.001 |  |
| high | 5759 | 848 | 1 [Ref] | 0.98(0.74-1.29) | 2.20(1.08-4.45) | 1.86(1.02-3.38) | 1.05(1.02-1.08) |  |
| P value |  |  |  | 0.859 | 0.03 | 0.044 | 0.002 |  |
| Dietary fiber intake | |  |  |  |  |  |  |  |
| low | 5745 | 1053 | 1 [Ref] | 1.27(0.98-1.64) | 1.75(1.12-2.73) | 2.66(1.68-4.21) | 1.06(1.04-1.09) | 0.148 |
| P value |  |  |  | 0.072 | 0.014 | <0.001 | <0.001 |  |
| high | 5771 | 789 | 1 [Ref] | 1.24(0.89-1.72) | 2.39(0.92-6.22) | 1.80(0.90-3.61) | 1.05(1.01-1.09) |  |
| P value |  |  |  | 0.197 | 0.073 | 0.097 | 0.008 |  |
| Dietary cholesterol intake | |  |  |  |  |  |  |  |
| low | 5743 | 874 | 1 [Ref] | 1.04(0.76-1.41) | 2.05(1.11-3.76) | 1.36(0.82-2.26) | 1.04(1.01-1.07) | 0.017 |
| P value |  |  |  | 0.814 | 0.022 | 0.225 | 0.005 |  |
| high | 5773 | 968 | 1 [Ref] | 1.54(1.19-1.99) | 1.93(1.18-3.17) | 5.21(3.06-8.85) | 1.09(1.07-1.12) |  |
| P value |  |  |  | 0.002 | 0.01 | <0.001 | <0.001 |  |
| Caffeine intake | |  |  |  |  |  |  |  |
| low | 5750 | 945 | 1 [Ref] | 1.13(0.86-1.50) | 2.15(1.26-3.67) | 3.09(1.73-5.53) | 1.07(1.04-1.09) | 0.791 |
| P value |  |  |  | 0.371 | 0.006 | <0.001 | <0.001 |  |
| high | 5766 | 897 | 1 [Ref] | 1.28(0.95-1.74) | 1.90(1.11-3.24) | 2.05(1.26-3.34) | 1.06(1.03-1.08) |  |
| P value |  |  |  | 0.106 | 0.02 | 0.005 | <0.001 |  |
| CVD, cardiovascular disease; Ref, reference; PIR, family income-poverty ratio; BMI, body mass index; eGFR, estimated glomerular filtration rate; HEI, healthy eating index.  Model 3: adjustments for age, sex, race/ethnicity, education level, marital status, PIR, smoking status, alcohol consumption, BMI, HEI, disease histories (trouble sleeping, hypertension, diabetes, dyslipidemia, and cancer), blood pressure, glycohemoglobin, low-density lipoprotein, and eGFR.  P value for interaction: interaction of stratified variable and PHQ-9 score on CVD.  Low dietary intake was defined as a participant's dietary intake below the median of the included population. High dietary intake was defined as a participant's dietary intake above the median of the included population. | | | | | | | | |
