# Supplementary material for: Factors influencing the association between depressive symptoms and cardiovascular disease in US population
Source: Sci Rep. 2024 Jun 13;14:13622. doi: 10.1038/s41598-024-64274-3 (PMC11176288; doi:10.1038/s41598-024-64274-3)
Supplement: Supplementary file 6 — Supplementary Table 6. [file 41598_2024_64274_MOESM6_ESM.docx]

**Supplementary table 6. Results of sensitivity analysis.**

| Outcomes | Total | Cases | Depressive Status | | | | PHQ-9 as a continuous variable |
| --- | --- | --- | --- | --- | --- | --- | --- |
|  |  |  | No/Minimal | Mild | Moderate | Moderately severe/Severe |  |
| NHANES 2007-2014 | 9193 | 1475 | 1 [Ref] | 1.33(1.03-1.71) | 2.31(1.45-3.69) | 2.43(1.58-3.74) | 1.07(1.05-1.09) |
| P value |  |  |  | 0.031 | <0.001 | <0.001 | <0.001 |
| NHANES 2007-2012 | 6681 | 1099 | 1 [Ref] | 1.16(0.86-1.57) | 1.86(1.21-2.92) | 2.79(1.59-4.89) | 1.07(1.04-1.10) |
| P value |  |  |  | 0.321 | 0.007 | 0.001 | <0.001 |
| Covariate modification (alcohol consumption) | 11516 | 1842 | 1 [Ref] | 1.25(1.01-1.54) | 1.94(1.31-2.89) | 2.40(1.63-3.53) | 1.06(1.04-1.08) |
| P value |  |  |  | 0.037 | 0.001 | <0.001 | <0.001 |
| Covariate modification (total cholesterol) | 11516 | 1842 | 1 [Ref] | 1.29(1.05-1.58) | 2.04(1.36-3.06) | 2.48(1.65-3.71) | 1.06(1.04-1.08) |
| P value |  |  |  | 0.017 | <0.001 | <0.001 | <0.001 |
| Covariate modification (triglyceride) | 11516 | 1842 | 1 [Ref] | 1.26(1.02-1.55) | 1.93(1.32-2.83) | 2.36(1.65-3.39) | 1.06(1.04-1.08) |
| P value |  |  |  | 0.035 | 0.001 | <0.001 | <0.001 |
| Covariate modification (dietary protein intake) | 11516 | 1842 | 1 [Ref] | 1.26(1.02-1.56) | 1.97(1.31-2.97) | 2.37(1.60-3.51) | 1.06(1.04-1.08) |
| P value |  |  |  | 0.03 | 0.002 | <0.001 | <0.001 |
| Covariate modification (dietary sugar intake) | 11516 | 1842 | 1 [Ref] | 1.25(1.01-1.54) | 1.99(1.33-2.98) | 2.42(1.63-3.60) | 1.06(1.04-1.08) |
| P value |  |  |  | 0.04 | 0.001 | <0.001 | <0.001 |
| Covariate modification (dietary fiber intake) | 11516 | 1842 | 1 [Ref] | 1.25(1.01-1.54) | 1.98(1.32-2.96) | 2.40(1.63-3.56) | 1.06(1.04-1.08) |
| P value |  |  |  | 0.039 | 0.001 | <0.001 | <0.001 |
| Covariate modification (dietary cholesterol intake) | 11516 | 1842 | 1 [Ref] | 1.25(1.02-1.55) | 1.98(1.31-2.97) | 2.38(1.59-3.55) | 1.06(1.04-1.08) |
| P value |  |  |  | 0.035 | 0.002 | <0.001 | <0.001 |
| Covariate modification (caffeine intake) | 11516 | 1842 | 1 [Ref] | 1.25(1.01-1.54) | 1.99(1.33-2.98) | 2.43(1.63-3.63) | 1.06(1.04-1.08) |
| P value |  |  |  | 0.041 | 0.001 | <0.001 | <0.001 |
| Ref, reference; PIR, family income-poverty ratio; BMI, body mass index; eGFR, estimated glomerular filtration rate; HEI, healthy eating index.  Model 3: adjustments for age, sex, race/ethnicity, education level, marital status, PIR, smoking status, alcohol consumption, BMI, HEI, disease histories (trouble sleeping, hypertension, diabetes, dyslipidemia, and cancer), blood pressure, glycohemoglobin, low-density lipoprotein, and eGFR.  Covariate modification (alcohol consumption) defines alcohol consumption as no less than 1 drink/day for women and no less than 2 drink/day for men.  Covariate modification (total cholesterol) replaces low-density lipoprotein with total cholesterol as the covariate included in the model.  Covariate modification (Triglyceride) replaces low-density lipoprotein with triglyceride as the covariate included in the model.  Covariate modification (dietary protein intake) replaces HEI with dietary protein intake as the covariate included in the model.  Covariate modification (dietary sugar intake) replaces HEI with dietary sugar intake as the covariate included in the model.  Covariate modification (dietary fiber intake) replaces HEI with dietary fiber intake as the covariate included in the model.  Covariate modification (dietary cholesterol intake) replaces HEI with dietary cholesterol intake as the covariate included in the model.  Covariate modification (caffeine intake) replaces HEI with caffeine intake as the covariate included in the model. | | | | | | | |
